# Trees, terraces and llamas: Resilient watershed management and sustainable agriculture the Inca way

**DOI:** 10.1007/s13280-024-02121-5

**Published:** 2025-01-22

**Authors:** Michael R. Frogley, Alex Chepstow-Lusty, Graham Thiele, Constantino Aucca Chutas

**Affiliations:** 1 Department of Geography, University of Sussex, Brighton, BN1 9QJ UK; 2 CGIAR Research Program On Roots Tubers and Banana (RTB), International Potato Center, Lima, Peru; 3 ECOAN, Pasaje Navidad U-10, Urb. Ttio, Wanchaq, Cusco, Peru

**Keywords:** Andes, Climate change, Climate-smart agriculture, Environmental history, Inca, Resilient watershed management

## Abstract

The Inca and their immediate predecessors provide an exceptional model of how to create high-altitude functional environments that sustainably feed people with a diversity of crops, whilst mitigating erosion, protecting forestry and maintaining soil fertility without the need for large-scale burning. A comparison is provided here of landscape practices and impacts prior to and after the Inca, derived from a unique 4200-year sedimentary record recovered from Laguna Marcacocha, a small, environmentally sensitive lake located at the heart of the Inca Empire. By examining ten selected proxies of environmental change, a rare window is opened on the past, helping to reveal how resilient watershed management and sustainable, climate-smart agriculture were achieved. We contend that, in the face of modern environmental uncertainty, a second climate-smart agricultural revolution is necessary, but one that accounts for the significant social capital of highland communities whilst still leaning heavily on native crops, trees and livestock.

**Supplementary Information:**

The online version contains supplementary material available at 10.1007/s13280-024-02121-5.

## Introduction

Rural communities occupying montane landscapes, most often characterised by small-holder agrarian and pastoral economies, have always been vulnerable to multiple environmental and socio-economic stressors (e.g. Morton 2007; Lindner and Pretzsch 2012; Keleman Saxena et al. 2016; Altea 2020). These include structural features such as physical isolation and occupying a predominantly vertical landscape that is difficult to work, as well as shocks such as unpredictable weather patterns, water management issues (e.g. Mark et al. 2010; Makino et al. 2021) and variable exposure to agricultural policies and unpredictable markets (e.g. Morton 2007). Whilst vulnerable to such shocks, these small-holder rural livelihoods can nevertheless be made sustainable, in the sense of balancing closely interrelated environmental and socio-economic drivers whilst ensuring long-term food security (Altieri 2004; Plekhov et al. 2024), because indigenous agriculturalists are not passive actors and over many centuries have applied adaptive strategies to realise the potential of their landscapes (e.g. Smit and Wandel 2006; de la Riva et al. 2013; Hunter and Huamán Mesía 2023). Such agriculturalists have always based their livelihoods around understanding the concepts of risk management, combining historical ‘traditional’ knowledge with the development of adaptation strategies that attempt to mitigate constantly shifting conditions. Some argue that this is essentially an expression of what is now called climate-smart agriculture (CSA), whereby practices are adopted that sustainably improve agricultural productivity and system robustness (including food security and resilient watershed management) by adapting to prevailing climate change in a way that manages and maintains healthy ecosystems (e.g. Thornton et al. 2018).

Good examples of the adaptation strategies of indigenous agriculturalists have been played out in the central Andes of South America, an incredibly diverse region in terms of latitudinal climate variability and associated ecosystem differences across a severe topography. For at least the last two millennia, agriculturalists exploiting the highland Andean landscapes were able to develop sustainable practices that flourished over long time periods. A particular challenge across the region would have been the relatively rapid change in conditions during the Medieval Climate Anomaly (MCA, *c*. CE 1000–1200; Lüning et al. 2019), stimulating agriculturalists to adapt. Populations moved up valleys in response to climatic warming, expanded maize agriculture from mid-elevations up to *c*. 3400 m, shifted potato cultivation to even higher elevations and diversified crop varieties (National Research Council 1989; Zimmerer 1993; Bauer 2004). These tactics were often accompanied by the construction of large-scale terraces and associated irrigation infrastructure, the adoption of agroforestry techniques (Chepstow-Lusty and Winfield 2000), whilst at the same time practising controlled burning of landscapes and efficient animal husbandry involving native camelids (llamas and alpacas). These strategies all led to the stabilisation of landscapes, permitting topographical exploitation and the promotion and maintenance of soil health, enabling the sustainable generation of modest agricultural surpluses. This combination of food production, soil conservation and the protection of natural biodiversity continued to be developed and refined in the Andes during Inca times (*c*. CE 1400–1533), within the framework of an imposed dual agricultural economy. In this system, a limited number of crops (predominantly specific varieties of maize and, to a lesser degree, potatoes) were cultivated in state fields as tribute to the ruling Inca, with the needs of local populations being met by subsistence agriculture from a more biodiverse range of crops on their own small plots (National Research Council 1989; Zimmerer 1993; Kosiba 2018). Indeed, customs that related to ensuring food security became deeply interwoven with cultural values over time, including the practice of tribute (taxation), an expansion of a gift economy and the legal protection of certain trees (e.g. Sherbondy 1986; Chepstow-Lusty and Winfield 2000). Today we would recognise these close links between nature and culture as exemplifying socio-ecological production (sensu lato ‘Satoyama’-type) landscapes (Brown and Mitchell 2000; Natori and Hino 2021).

The largest external shock to the region was undoubtedly the invasion of the Spanish in the sixteenth century, when diseases (including measles, smallpox and influenza) and, to a lesser extent, conflict, decimated native populations (Kosiba and Hunter 2017). It has been estimated that the Inca elite may have overseen a population of between 6 and 14 million (McEwan 2006), yet within a century of the Spanish Conquest, the indigenous population in Peru had shrunk to about 600 000 (Cook 1981). Additional shocks included the introduction by the Spanish of new landscape management practices, new crops and new livestock (many of which were often detrimental to the landscape), along with increased pressure for tribute, mass resettlement and labour-draft. Although the local economies initially adapted to these changes and even generated agricultural surpluses in some regions (Wernke and Whitmore 2009; Hunter and Huamán Mesía 2023), over time and with increased socio-economic pressures associated with significant demographic decline and climatic shocks, predominantly the Little Ice Age (LIA), agriculture de-intensified and landscapes became degraded (Wernke 2010). Many terrace and irrigation systems were abandoned and fell into disrepair (Denevan 1986, 2001; Hunter 2021); deforestation led to reduced watershed protection and degradation of soil health, whilst adoption of non-native livestock (e.g. cattle, sheep and goats) was highly detrimental to fragile soils and landscapes. This legacy has led to the increased vulnerability of highland landscapes and rural economies to present-day external shocks, related to ongoing climate change and the associated increased intensity and variability of adverse weather systems.

Climate change is now occurring at faster rates than experienced in the past (Anderson et al. 2011) and the World Bank suggests that Peru is one of the countries most vulnerable to climate shock and water stress in Latin America (World Bank 2015). These pressures are already negatively impacting Andean agricultural systems and food security (Mark et al. 2010; Lindner and Pretzsch 2012; Crespo-Pérez et al. 2015; Thiele et al. 2017; Tito et al. 2018; Vidal Merino et al. 2020). Notwithstanding that ‘sustainable agriculture’ is a concept grounded ultimately by socio-political and environmental expediency (Kaptijn 2018; Plekhov et al. 2024), it has nevertheless been suggested that pre-Colonial agricultural and land management practices might point the way for *contemporary* climate-smart approaches to restore landscapes and adapt livelihoods to help mitigate future impacts (Chepstow-Lusty et al. 2009; Thiele et al. 2018).

Much of the literature around this topic concentrates either on the environmental component (mostly external challenges due to climatic drivers) or the socio-political component (mostly internal challenges wrought by what is perceived as poor political and/or economic practices). Here, we try to combine both perspectives by using an unusually detailed and continuous palaeoenvironmental record from Marcacocha, a wetland site that reflects the history of a small watershed in the heart of the central Andean highlands. The sedimentary record from Marcacocha has not only recorded how climate has changed over the past few centuries but has also documented how agriculturalists have adapted during both pre-Colonial and Colonial times.

## The setting: the Marcacocha basin

**Fig. 1:**
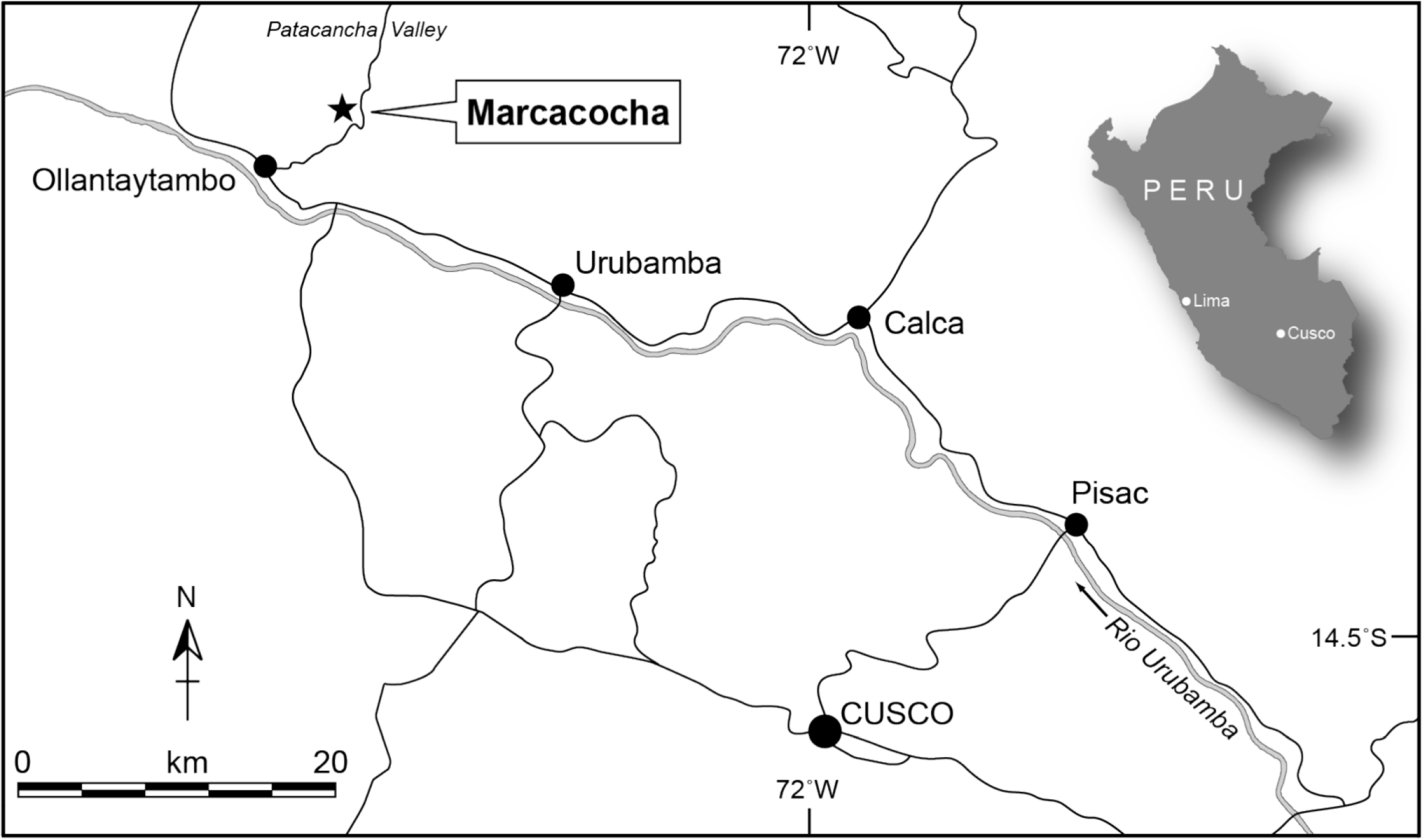
Location map showing Marcacocha in the Cusco region and other major sites (adapted from Chepstow-Lusty et al. 2009)

**Fig. 2:**
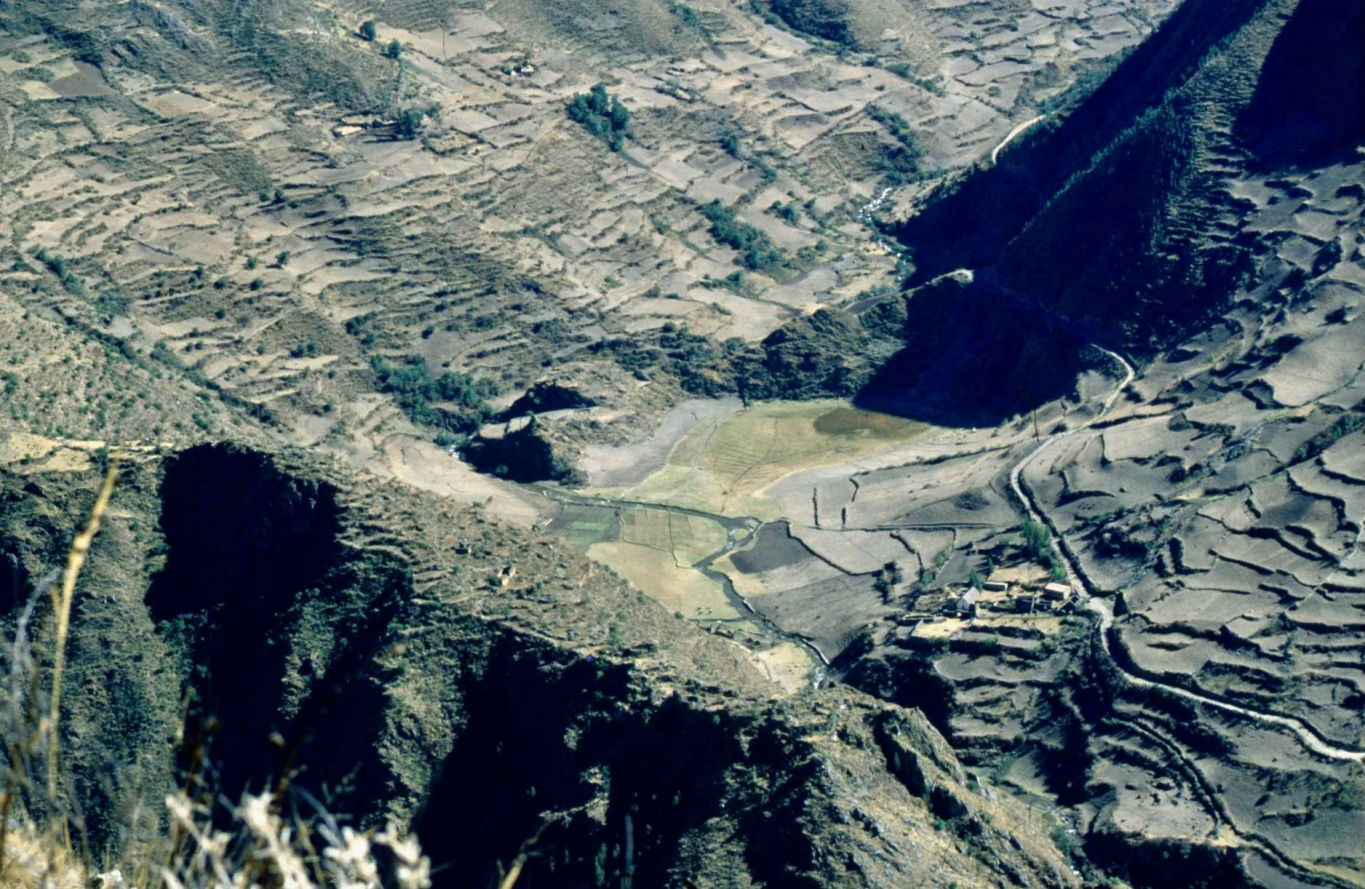
View looking northwards upstream over the Patacancha Valley, with the small infilled lake of Marcacocha (defined by the dark circle of sedge vegetation) set within a landscape of terraces, under-utilised fields and archaeological remains. Photograph taken August 1996 with Marcacocha partly in shadow before 8.30 a.m (Photo by A. Chepstow-Lusty)

The Marcacocha basin (13°13′S, 72°12′W; 3355 m above sea-level) is a discrete area of grassland pasture in the Patacancha Valley, a minor tributary of the Urubamba River in the central Andean highlands of Peru, located approximately 45 km to the north-west of the regional capital of Cusco (Fig. 1). Situated within the pasture is a circular area of wetland < 40 m diameter, which represents the infilled remnants of a small, former lake (Laguna Marcacocha). The present-day wetland is separated from the Patacancha River by the Huchuy Aya Orqo ridge (*c*. 100 m to the west), which forces the river to make a loop around the western margin of the basin (Fig. 2). This ridge contains archaeological deposits that range from the Early Horizon Period (*c*. 800 BCE) to the Inca period (Kendall and Chepstow-Lusty 2006); the surrounding landscape is extensively anthropogenically modified, being densely covered in underused and poorly maintained irrigation systems and agricultural terraces of Inca and pre-Inca origin (Fig. 2). The density of ruins and groundworks are an indication that this landscape was engineered to support populations beyond those local to the basin. Indeed, during the Inca period Marcacocha served the royal estate that had been established in the fertile landscapes centred around the monumental town of Ollantaytambo (Kosiba and Hunter 2017; Kosiba 2018), situated approximately 10 km downstream at the confluence of the Patacancha and the Urubamba rivers (Fig. 1). Kendall (1991) estimated that almost 2400 hectares of Inca and late pre-Inca terraces were evident across the Ollantaytambo district, perhaps supporting *c*. 100 000 non-local people. Whereas pre-Inca people used landscape modification for soil conservation and agricultural reasons, the Incas appear to have also used it as part of a major strategy for food security at a time of empire building, population growth and redistribution.

## Materials and methods

A stratigraphically continuous 8.25 m sequence of sediments was recovered from the centre of the wetland in 1993 using a Livingstone corer and subjected to high-resolution palaeoecological analysis (Chepstow-Lusty et al 1996, 1998, 2003). Chronological control of the topmost 6.3 m of organic sediments, provided by a combination of seven ^210^Pb and six radiocarbon dates (Table S1), suggests that the sequence extends back for *c*. 4200 years (Chepstow-Lusty et al. 2009). Detailed, multi-proxy analysis of the sediments at decadal to sub-decadal resolution shows that the lake responded sensitively to environmental and anthropogenic changes impacting the basin over this time (Chepstow-Lusty et al. 2003, 2007, 2009, 2019; Sterken et al. 2006). As a result, the sequence has captured changes in agricultural practices and landscape use throughout the occupation of the basin. This not only includes the early development of pastoralism in the region, but also the increasing cultivation and intensive management of the landscape under successive cultures, including the imperial phase of the Inca (*c*. CE 1400–1533). Crucially, the record also captures the dramatic transformation of the region following the arrival of the Spanish and highlights the subsequent broad de-intensification of agricultural practices and degradation of the landscape (up until the final infilling of the lake in the early nineteenth century).

**Fig. 3:**
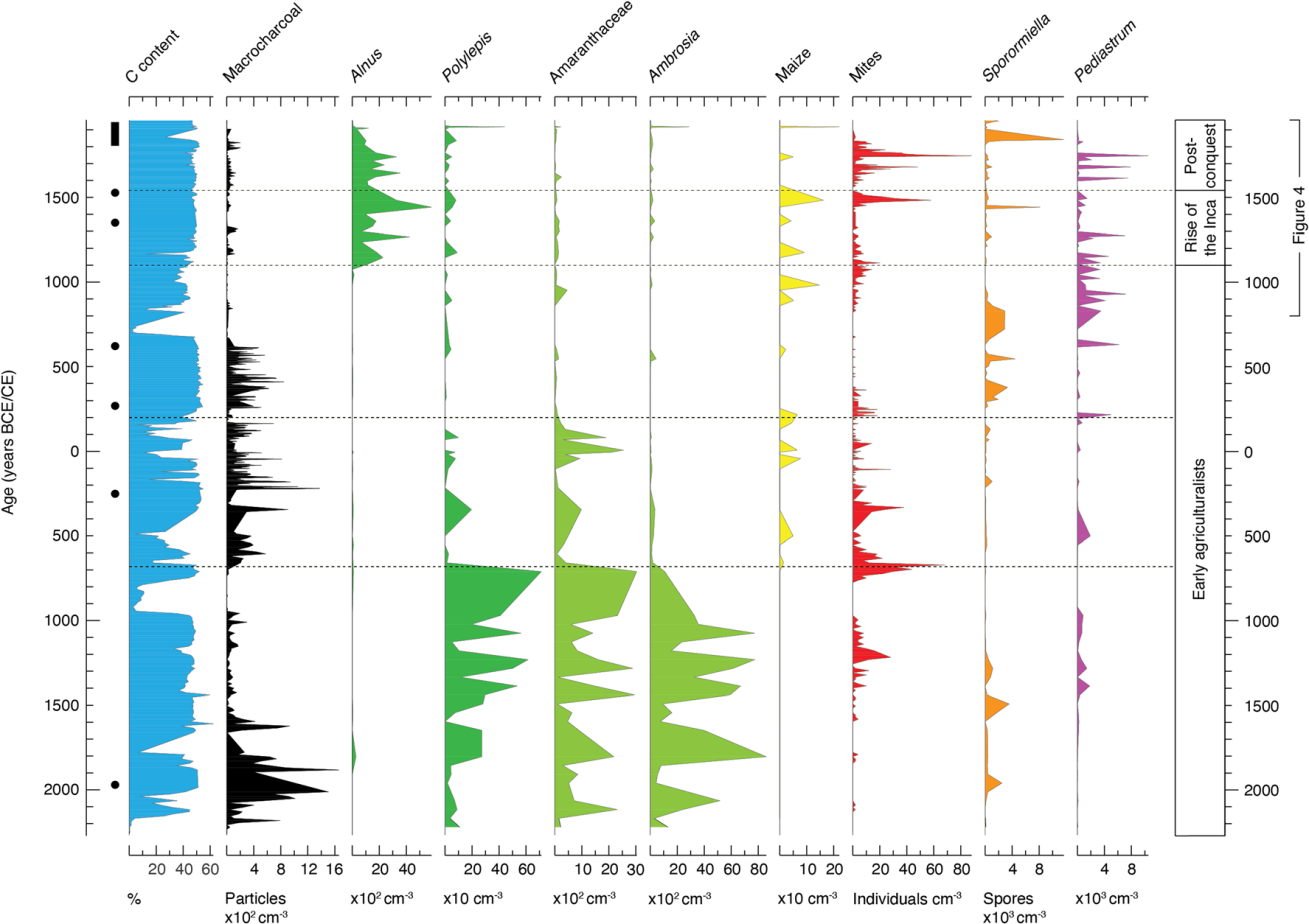
Selected proxies from the lake sediments of Marcacocha highlighting land-use and other environmental changes during the past 4200 years. Radiocarbon dates are denoted by filled circles; the range of ^210^Pb dates is denoted by the filled rectangle (Table S1). Dashed horizontal lines and right-hand column highlight historical periods discussed in the text

Whilst the 4200-year Marcacocha record contains multiple geochemical, biological and physical proxies, the main narrative is summarised here by the use of ten key palaeoecological indicators, selected to best illustrate environmental changes and shifts in land-use practices (Table 1; Fig. 3). Concentrations of the algae *Pediastrum* constitute the only totally new dataset to the Marcacocha record presented here. Algal spore counts were obtained from the same slides/residues from which the pollen abundances were derived (see Chepstow-Lusty et al. 2003 for the full methodology). Similarly, only the past 1200 years of the macrocharcoal and *Sporormiella* data have hitherto been published.

**Table 1:** Key palaeoenvironmental proxies

Indicator		Characteristics
1	Sedimentary carbon content	Low percentages may indicate erosion in the immediate landscape and may be exacerbated by human activity and/or aridity
2	Macrocharcoal concentration	Charcoal particles > 125 µm diameter. Indicates local landscape burning both in and around the basin
3	*Alnus* pollen concentration	Alder is an abundant, wind-pollinated tree. The only alder native to S. America is *A. acuminata*, a nitrogen-fixing colonising species that grows rapidly and produces a relatively straight trunk
4	*Polylepis* pollen concentration	Native to S. America, this is the highest arboreal genus in the world. Its pollen is dispersed only short distances (its presence therefore indicates local populations); it is poorly adapted to fire
5	Amaranthaceae pollen concentration	A group of plants adapted to dry-cold conditions, producing many edible wild and domesticated species including quinoa (*Chenopodium quinoa*)
6	*Ambrosia* pollen concentration	A ruderal species (often colonising eroded soil), this can be a sizeable shrub. *A. arborescens* is the only species of *Ambrosia* found in this region
7	Maize pollen concentration	One of the most important Andean crops. Pollen grains are large (commonly > 70 µm diameter) which limits transportation and/or preservation. Generally indicative of local cultivation
8	*Hydrozetes* mite concentration	An aquatic oribatid mite taxon that feeds on plant detritus and broken-down dung. In situ populations can be indicative of localised livestock presence
9	*Sporormiella* dung fungal spore concentration	Often taken to indicate the presence of livestock. Due to their restricted dispersal, coprophilous spore concentrations can also be indicative of lake-level changes (Raper and Bush 2009)
10	*Pediastrum* algae concentration	More correctly *Pediastrum boryanum*/*duplex* algae. Can be indicative of water quality, with higher concentrations suggesting elevated nutrient availability and/or localised erosion, for example, linked to the presence of livestock

## Results and interpretation

### Early agriculturalists at Marcacocha (*c.* 2200 BCE–CE 1100)

Following the ‘early–mid-Holocene climatic optimum’ *c*. 8000–4000 years ago (a period characterised by aridity in the Central Andes), conditions gave way to a sustained wetter period during which the small circular lake of Marcacocha formed and began to accumulate organic sediments. In the early part of the record (*c*. 2200 BCE–*c*. 700 BCE), low concentrations of *Polylepis* pollen suggest that conditions were (initially at least) relatively dry and cool, although there is clear evidence to indicate that the lake and surrounding pasture were nevertheless resources that were being exploited during the uneven transition from hunter-gatherer to early agriculturalist livelihoods (Fig. 3). Evidence for early human activity in the basin comes from high concentrations of Amaranthaceae pollen (likely including relatives of quinoa and other early domesticates) and the presence of livestock (*Sporormiella* and mite concentrations); furthermore, the macrocharcoal, carbon content and *Ambrosia* data collectively suggest that the landscape was prone to erosion and burned on a frequent basis, to which *Polylepis* was particularly vulnerable.

The first occurrence of maize pollen at around 700 BCE, coupled with the marked decline of Amaranthaceae and *Ambrosia* pollen, not only suggests the adoption of more structured and sedentary agricultural practices, including weeding (also seen more widely across the region; e.g. Bruno and Whitehead 2003), but also indicates a major climatic shift towards more favourable warmer and wetter conditions (Fig. 3). Burning of the landscape continued to be a widespread practice (likely to clear ground and maintain soil fertility), with significant declines in the remaining *Polylepis* cover suggesting that woodland resources were not being carefully managed. The increase in *Hydrozetes* oribatid mite concentrations and sustained coprophilous fungal spore presence indicates that livestock continued to use the lake pasture (and may also have increased in numbers), though the muted spore record suggests that the wetter climate had resulted in higher lake levels (the spores have a limited dispersal range, indicating that the distance of the shore from the core site had probably increased; see Raper and Bush 2009).

The return of much cooler and drier conditions from around CE 200 and an associated decline of activity on the landscape is mirrored by local and regional archaeological and palaeoclimatic records (e.g. Johannessen and Hastorf 1990; Seltzer and Hastorf 1990; Kendall and Chepstow-Lusty 2006). Nevertheless, major burning continued around the basin until *c*. CE 700 when it declined markedly, possibly due to increasingly arid conditions and a reduction in the vegetation that could be sustained on the landscape after then (Fig. 3). Lake-levels were likely low at this time; the algal *Pediastrum* record suggests elevated levels of nutrients entering the lake, probably derived from livestock which had a presence in the basin (Chepstow-Lusty 2011).

Around CE 600, the Wari culture expanded into the Cusco region from the north (Bauer 2004), where they remained until drought conditions likely led to a rapid population decline around four centuries later. Although they mostly occupied lower altitude sites, there is evidence for some maize agriculture in the vicinity of the basin, suggesting that conditions improved from time to time (Chepstow-Lusty et al. 2003, 2009).

### Altitudinal migration and the rise of the Inca (***c.*** CE 1100–1540)

**Fig. 4:**
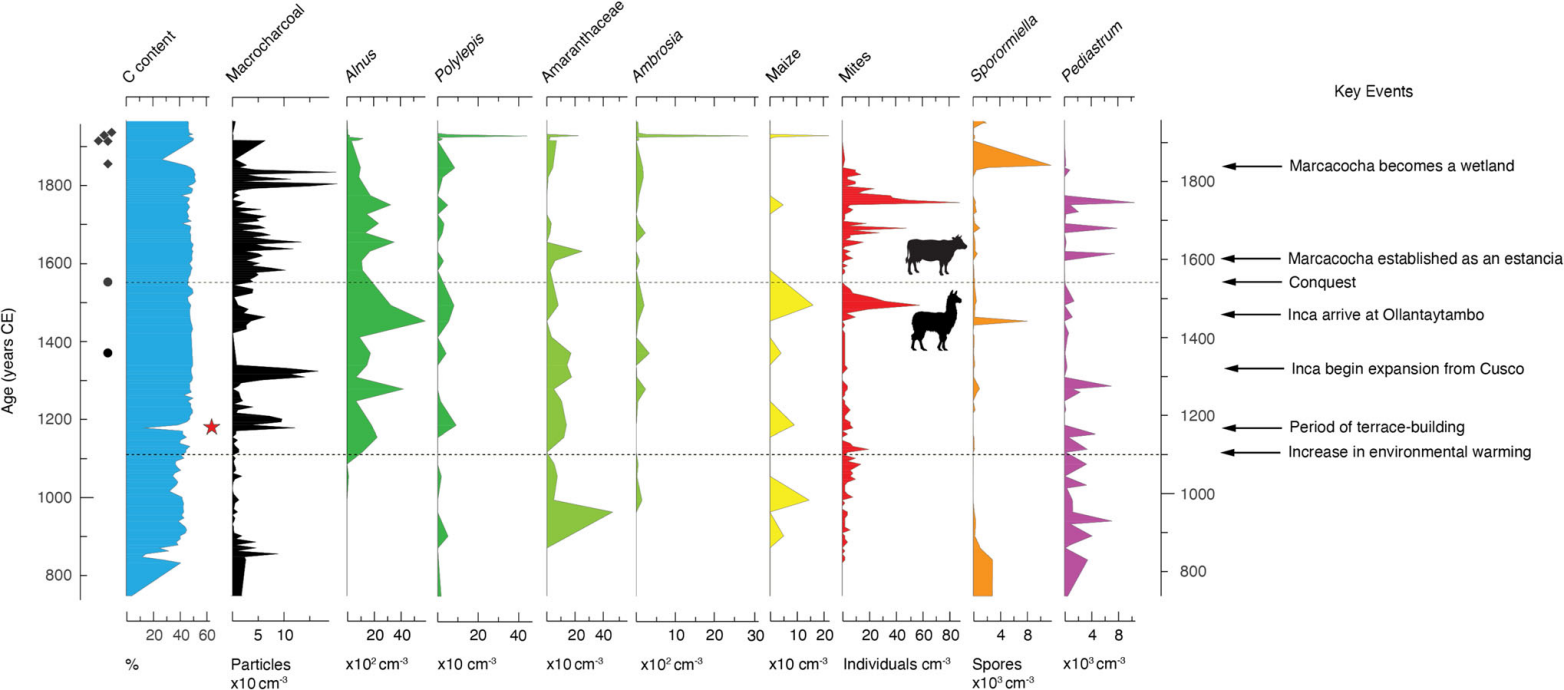
Selected proxies from the lake sediments of Marcacocha highlighting land-use, other environmental changes and key historical events over the past 1200 years. Radiocarbon dates are denoted by filled circles and ^210^Pb dates by filled diamonds (Table S1). The red star indicates the decline in per cent carbon during the deposition of a thin, inorganic layer consisting of hard shaley material and interpreted as being associated with the construction of terracing in the basin. Silhouettes denote the switch away from traditional camelid livestock to animals introduced and favoured by the Spanish, such as cattle, sheep, goats and horses

From around CE 1100, the region experienced a sustained increase in warming, most probably part of the broader expression of the Medieval Climate Anomaly across South America, *c*. CE 1000–1200 (Lüning et al. 2019). The warming climate allowed vegetation to grow at higher altitudes than hitherto; evidence for this biological vertical migration is seen most strikingly at Marcacocha by the expansion of alder (*Alnus acuminata*) on the slopes around the basin, a relatively fast-growing tree that favours moister conditions that would have been provided by the perennial meltwater flow of the Patacancha River (Fig. 4). Local archaeological evidence confirms that agriculturalists followed this upward migration and expanded their agropastoral activities in the basin (Kendall and Chepstow-Lusty 2006). This is echoed by the palaeoecological evidence, which indicates cultivation of maize and increased livestock presence (in turn influencing the influx of nutrients to the lake and accounting for the elevated concentrations of *Pediastrum* algae seen in the sedimentary record; Fig. 4).

At around CE 1200, a thin, inorganic layer consisting of hard shaley material was deposited in the lake (Fig. 4). This is interpreted as evidence for major groundworks related to the construction of terraces and other field systems around Marcacocha, which stabilised the landscape, conserved soils and significantly reduced levels of erosion across the basin (in part evidenced by consistently suppressed levels of algal *Pediastrum*). The timing of this work, at least two centuries before the expansion of the Inca from their Cusco heartland and after the decline of the Wari culture, suggests that this was likely an example of local community-led environmental innovation, rather than something imposed by a ruling elite (Janusek and Kolata 2004; Langlie 2018).

**Fig. 5:**
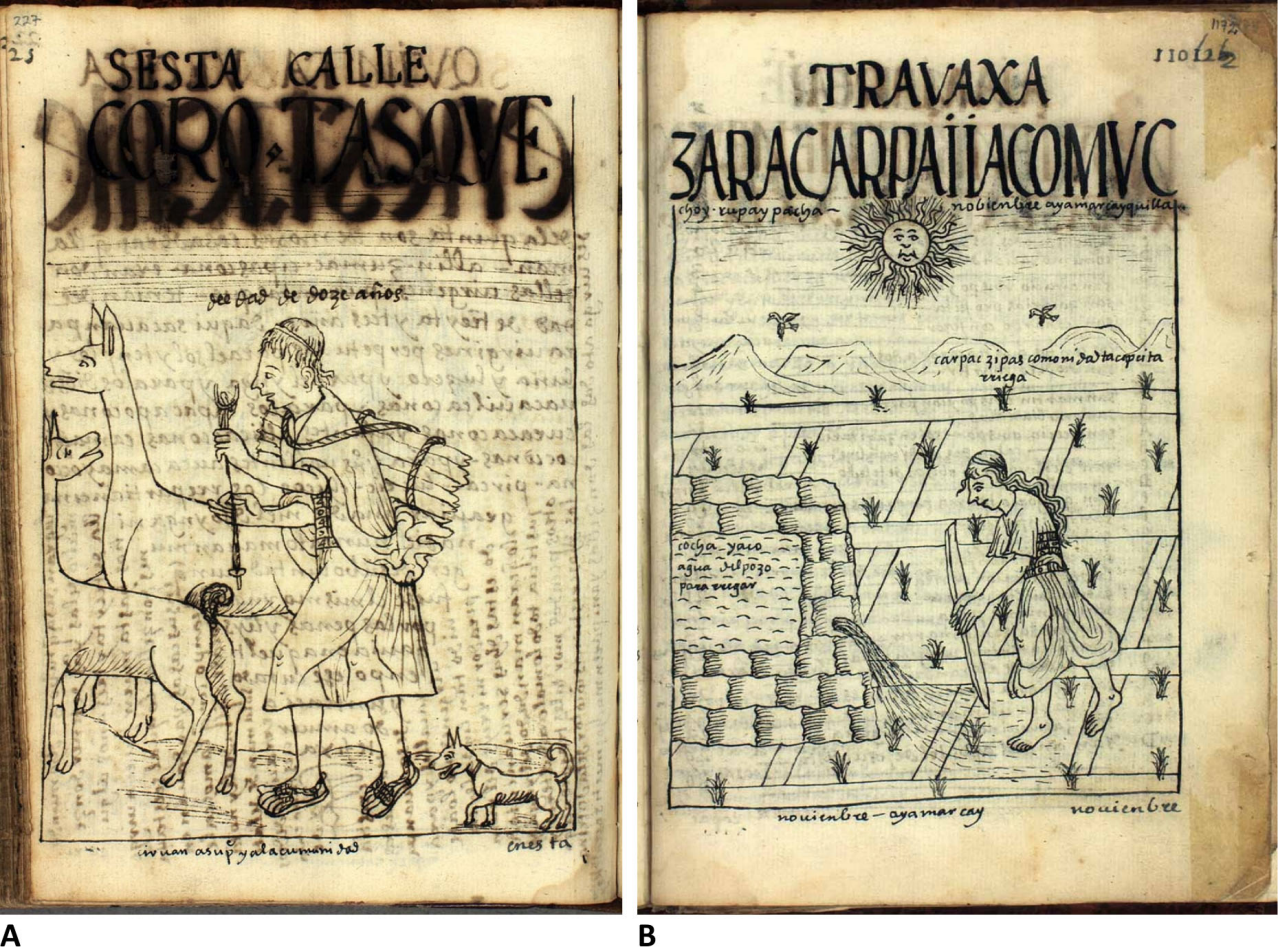
Etchings by Guaman Poma *c*. AD 1615. **A** ‘Short-haired girl of 12 years age’ (translation), carrying a fardo of wood and herding llamas. **B** ‘November: Time of watering the maize, of scarcity of water, time of heat’ (translation), showing irrigation of field systems. Images: Royal Danish Library, GKS 2232 quarto: Guaman Poma (*c*. 1615), *Nueva corónica y buen gobierno*, pages 227 (**A**) and 1172 (**B**)

In the early fourteenth century, the Inca state began an aggressive campaign of expansion from the Cusco region (Bauer 2004), ultimately establishing a trans-Andean empire that stretched from the modern-day southern Colombian border to central Chile. Arriving at Ollantaytambo *c*. CE 1450, they swiftly established a royal estate (Kosiba and Hunter 2017), from where they began to control the region. Although Marcacocha was located above the main estate itself, Incan tributary labour demands meant that the local agriculturalist population undertook intensive land and forestry management across a range of altitudes (Hunter 2021). It is well established that the Inca engaged in both agroforestry and the wider protection of woodland resources (e.g. Johannessen and Hastorf 1990; Chepstow-Lusty and Winfield 2000) and were accomplished in efficiently irrigating and maintaining terraced fields (Fig. 5). High concentrations of *Alnus acuminata* pollen in the sedimentary record (which reach their peak in this period), coupled with low levels of erosion (as indicated by reduced concentrations of algal *Pediastrum* and *Ambrosia arborescens* pollen) provides evidence of Inca agroforestry in this location (Chepstow-Lusty et al. 2003, 2009) (Fig. 4). Areas of pasture above royal estates were also commonly used to pasture camelid herds (Niles 1999), as reflected in the Marcacocha record by elevated livestock proxies (both oribatid mite and *Sporormiella* concentrations; Fig. 4). These values may also reflect the importance of the basin for trading purposes, since the basin is situated on an important long-distance trading route (probably originating from pre-Incan times) that links the highlands with the Amazonian selva to the east. Spanish sources suggest that during the late fifteenth and early sixteenth centuries, caravan trains of up to a thousand camelids would regularly pass through the Patacancha Valley and utilise the pasture surrounding the lake (de Acosta 1590; de la Vega 2014 [1609]); the local population would have facilitated support for the caravans and other travellers under their tributary labour obligations to the Inca (Hunter 2021).

### Post-conquest (***c.*** CE 1540 onwards)

The arrival of the Spanish was transformational, both societally and environmentally. In the decades following the invasion, the Inca estates were seized by the new colonial powers, who introduced hacienda-style land management and employed local residential workers to farm both native and introduced crops for commercial purposes, especially maize and wheat (Hunter 2021; Hunter and Huamán Mesía 2023). It is well known that diseases carried by the invaders decimated local populations and that the Spanish therefore engaged in forcible migration and consolidation of labour across the region (in part via the *reducción* settlements) in order to service the haciendas. Whilst maize continued to be cultivated at Marcacocha, tuber crops (requiring less irrigation) were increasingly grown at higher elevations (the basin is situated at the effective quechua/suni agricultural boundary defining the upper range of maize and lower limit of potato cultivation in the region). Demographic decline led to inevitable degradation and abandonment of non-irrigated and higher altitude terracing, the efficient maintenance of which is highly labour-intensive (Inbar and Llerena 2000; Denevan 1986, 2001). Interestingly, the macrocharcoal record from Marcacocha (Figs. 3 and 4) suggests that field-burning remained a feature on the landscape (albeit to a significantly lesser extent than in the past) and may reflect hacienda workers trying to clear overgrown agricultural land (Chepstow-Lusty et al. 2009).

Perhaps less well known is that disease also struck the native livestock in the mid-sixteenth century, immediately following the Spanish conquest; llama and alpaca herds were susceptible to skin diseases, known as carache or llama mange, which caused high levels of mortality (de Acosta 1590; de la Vega 2014). Rapid declines in oribatid mite concentrations in the Marcacocha sequence reflect this collapse in camelid presence around the lake (Chepstow-Lusty et al. 2019), whereas subsequent recovery in values half a century later almost certainly reflect shifts in animal husbandry practices (Fig. 4). Marcacocha was established as a Spanish estancia (ranch) at this time, with Old World livestock such as sheep, cows, goats and horses supplanting more traditional camelids (Hunter 2021). However, a combination of hooved, heavier-bodied animals on the landscape and poorly maintained groundworks contributed to erosion and destabilisation of the slopes around the basin. This is indicated most notably in the lake core sequence by peaks in algal *Pediastrum* concentrations, reflecting in part the significant negative impact of livestock waste on lake water quality (Fig. 4).

Also contributing to landscape degradation was sustained deforestation; evidence from the sequence at Marcacocha includes notable declines in alder and other indigenous species such as *Polylepis* (Figs. 4 and Fig. S1). This reflects the broader issues around the collapse of Inca-mediated regulation protecting forestry and the Spanish demand for tribute in the form of timber to meet construction and fuel requirements in the cities (Johannessen and Hastorf 1990; Chepstow-Lusty and Winfield 2000). Local households experienced significant fuel poverty in order to supply the onerous annual tribute (Wernke and Whitmore 2009) and there is evidence for a shift from woody fuels to a greater use of dried crop stalks and dung (Hunter and Huamán Mesía 2023). Indeed, accounts from the time (e.g. de Cobo 1639) noted that Spanish households were extremely wasteful, using as much fuel in a single day as an indigenous household would use in an entire month. However, it is notable that by the end of the sixteenth and beginning of the seventeenth centuries there was a limited resumption of agroforestry by the Spanish (Sherbondy 1986; Chepstow-Lusty and Winfield 2000; Sublette Mosblech et al. 2012).

### The last* c.* 200 years

In about CE 1800, Marcacocha ceased to be a lake, becoming instead a wetland dominated by totora sedge (*Schoenoplectus californicus* ssp. *tatora*) growing on top, accompanied by the subsequent deposition of peat (Fig. 2). This transition is clearly marked by the initial deposition of a hard inorganic band (low C %) of sediment (Fig. 4) and a high concentration of *Sporormiella* dung fungal spores (Chepstow-Lusty et al. 2003, 2019) suggesting that livestock could get closer to what had been the centre of the lake.

Although direct archiving of environmental and anthropogenic change at Marcacocha ceased with this final infilling, it is known from historical documents that the last couple of centuries have seen an acceleration of impacts and vulnerabilities. Continued demographic decline and subjugation of native labour by new landowners, changes in agrarian and land management practices, and a shift in agricultural emphasis to the more fertile valley-bottom areas, meant that the highland areas in particular experienced a slow agricultural de-intensification during Colonial times (e.g. Wernke 2010; Hunter 2021). Since the late nineteenth century, in the Cusco region and beyond, Andean tree species have most commonly been replaced by ecologically damaging, fast-growing *Eucalyptus* (Dickinson 1969; Gade 1975; Young and Lipton 2006; Fig. S1), except at the highest altitudes, where fragments of *Polylepis* woodland can still be found (Fjeldså and Kessler 1996; Fig. 4).

## Priorities: trees, terraces and camelids

In late January 2010, following two weeks of heavy rain and the establishment of a new long-term monthly precipitation maximum (Huggel et al. 2015), a state of emergency was declared in six provinces of the Cusco region and all of neighbouring Apurimac, with rivers bursting their banks, numerous landslides and major flooding. Lucre and Urubamba were inundated, whilst bridges in Pisac and Ollantaytambo were swept away. The Sacred Valley was only accessible by air (as was Machu Picchu), with the railway line cut in many places and thousands of tourists and locals having to be airlifted out to safety. It was estimated at the time that 25 000 people in the region were made homeless, whilst 80% of crops (including maize that was ready for harvesting) were wiped out; economic losses were estimated to be around $230 million (Huggel et al. 2015). Was the severity of the impact of this intense rainfall event a legacy of post-Colonial landscape decline, i.e. evidence that the underpinning environmental and socio-economic drivers of sustainability were out of balance, which had led to the abandonment of sustainable, traditionally rooted methods of land management?

Andean communities in the twenty-first century are facing unprecedented challenges. From an environmental perspective, recent decades have seen increased climate extremes (Anderson et al. 2011; Huggel et al. 2015) and an increase in warming trends (Vuille et al. 2008) coupled with a reduction in glacial extent (Bradley et al. 2006; Vuille et al. 2008; Mark et al. 2017). Peru is currently considered among the countries in South America most vulnerable to different impacts of climate change and has around 70% of the world´s tropical glaciers, vital for supplying water not only to the highlands, but also the arid coast, where the capital Lima and other major settlements are located (Bergmann et al. 2021). The glaciers are dependent on moisture generated from the Amazon basin, itself potentially threatened by die back, and it is estimated overall that Peruvian glaciers have shrunk by about 30% since 2000 (Seehaus et al. 2019). Similarly, in the Cordillera Vilcanota (of particular relevance here in the southern Peruvian Andes), there has been a marked ice loss since 1985, comprising about 30% by area and 45% by volume, respectively (Salzmann et al. 2013), reducing the capacity of the ice fields to buffer against climate variability.

Despite increased meltwater levels, poor watershed management, including increases in riverine and ground-water abstraction, has driven significant household declines in water availability in many areas (Mark et al. 2010). Higher average annual temperatures have exacerbated the occurrence of crop-based pests and diseases (Giraldo et al. 2010) and reduced the viability of some crops and driven shifts in cropping. For example, the cultivation of native potatoes has already experienced climate-related changes: in the central Andes they are now being grown at altitudes more than 300 m higher on average than in 1975 (Arce et al. 2019). These environmentally driven pressures have been coupled with an increased focus on market-led agricultural policies, which have in some cases driven the emergence of more diverse agricultural production strategies and opened up new opportunities (e.g. Radolf et al. 2022). Similarly, whilst migration of labour to the cities and the rise of tourism over the past few decades have in some ways contributed to increased livelihood vulnerability, these factors have at the same time been mitigated by the development of alternative income strategies (e.g. Steven et al. 2013; Romeo et al. 2021).

Warming trends and increased rainfall in the Andes are not new phenomena (although the rate of change may be greater than in the past). During the MCA, for example, the Marcacocha record demonstrates that communities in the Patacancha Valley moved up to higher altitudes as conditions warmed, colonising these new areas for agriculture in part due to the availability of increased meltwater. The strategies they employed included terracing of the landscape, maintenance and protection of natural forest cover and the strategic planting of native woodland at higher altitude and along watercourses. Acting together, these measures increased infiltration and retention (capturing and releasing water slowly and efficiently), reduced run-off (thereby minimising flooding and loss of soils due to erosion) and enhanced crop production (particularly maize and tubers, such as potatoes, but also permitting a wide diversity of crops to be grown at different altitudes; National Research Council 1989; Zimmerer 1993). This is resilient watershed management by any other name and it would seem plausible that managed (re)adoption of the modern equivalent of such techniques today could incentivise more widely environmentally beneficial climate-smart agricultural practices and their importance through policies in order to sustain the livelihoods of small-holder agriculturalists.

Some authorities regard the agricultural innovation of the Inca and their immediate predecessors as a climate-smart revolution for that time and have called for a second Andean revolution for the twenty-first century (e.g. Thiele et al. 2017, 2018; Quispe Conde et al. 2022; Arias Montevechio et al. 2023). Climate-smart agriculture involves practices that sustainably increase productivity and system resilience whilst reducing greenhouse gas emissions (FAO 2010). This approach would require a portfolio of strategies including diversification of crops with a focus on traditional, climate-resilient cultivars (Arias Montevechio et al. 2023) and maintenance of their genetic diversity to allow ongoing genetic adaption to climate change (e.g. Young and Lipton 2006), along with improved husbandry of climate-resilient livestock that impact the environment less, such as native camelids (e.g. López-i-Gelats et al. 2015). Hand-in hand with this would be an expansion of agroforestry and planting of indigenous tree species, especially at the higher altitudes. This latter strategy would recreate a more continuous cover of the high-altitude *Polylepis* woodlands, with *Escallonia* spp., *A. acuminata*, *Gynoxys* spp.*, Buddleia* spp*.* and other native species lower down, to both conserve water and to reduce surface run-off. Further to this, a limited return to terrace farming techniques would help to prevent soil erosion and improve soil fertility. However, given the enormous labour and resource requirements, a wide-scale rebuilding of terraces which have been abandoned in most locations is unlikely to be realistic (Plekhov et al. 2024). In particular, the returns from cultivating maize and annual crops are usually insufficient to cover the additional investment, notably of labour required for maintaining terraces in the long term. Nevertheless, communities could be encouraged to find more locally applicable solutions (Zimmerer 2000), which might include restoration of selective terracing and certain irrigation canals (Posthumus and Stroosnijder 2010), combined with agroforestry potentially linked to marketing of higher value crops or through tourism-linked incentives for landscape restoration (Londoño et al. 2017).

However, whilst there are no shortage of climate-smart strategies available to help highland rural communities build resilience, NGOs working with local communities on these schemes in the past have often met with limited enduring success (Bandy 2005; Kendall and Chepstow-Lusty 2006; Plekhov et al. 2024). In the Patacancha Valley, for example, the 6 km-long Pumamarca canal and surrounding terraces were restored in 1997 as part of a major international programme employing local people, but the works were not subsequently maintained and mostly abandoned shortly after completion (Kendall and Chepstow-Lusty 2006). Whilst not atypical, in part it is likely that these outcomes are a result of poor engagement with, and incentivisation of, local communities, driving a sense of insufficient empowerment and insufficient attention to higher market value opportunities or other incentives to maintain landscapes (e.g. Treacy 1989; Plekhov et al. 2024). Similarly, pastoralists across the central Andes recognise that native camelids impact the landscape far less than introduced livestock, are easier to rear and are more resilient to future environmental shifts. However, the market for llama-derived products in particular is limited and largely uneconomic (Radolf et al. 2022), forcing more diverse livestock production strategies. Viable climate-smart solutions therefore require collaboration with governmental and non-governmental entities to help empower Andean communities to make informed decisions about their livelihoods, developing market opportunities for native potatoes and indigenous animal species, as well as recognising the deep cultural importance of traditional knowledge and shared values (Fischer et al. 2012; Devaux et al. 2020; Barrera et al. 2021).

**Fig. 6:**
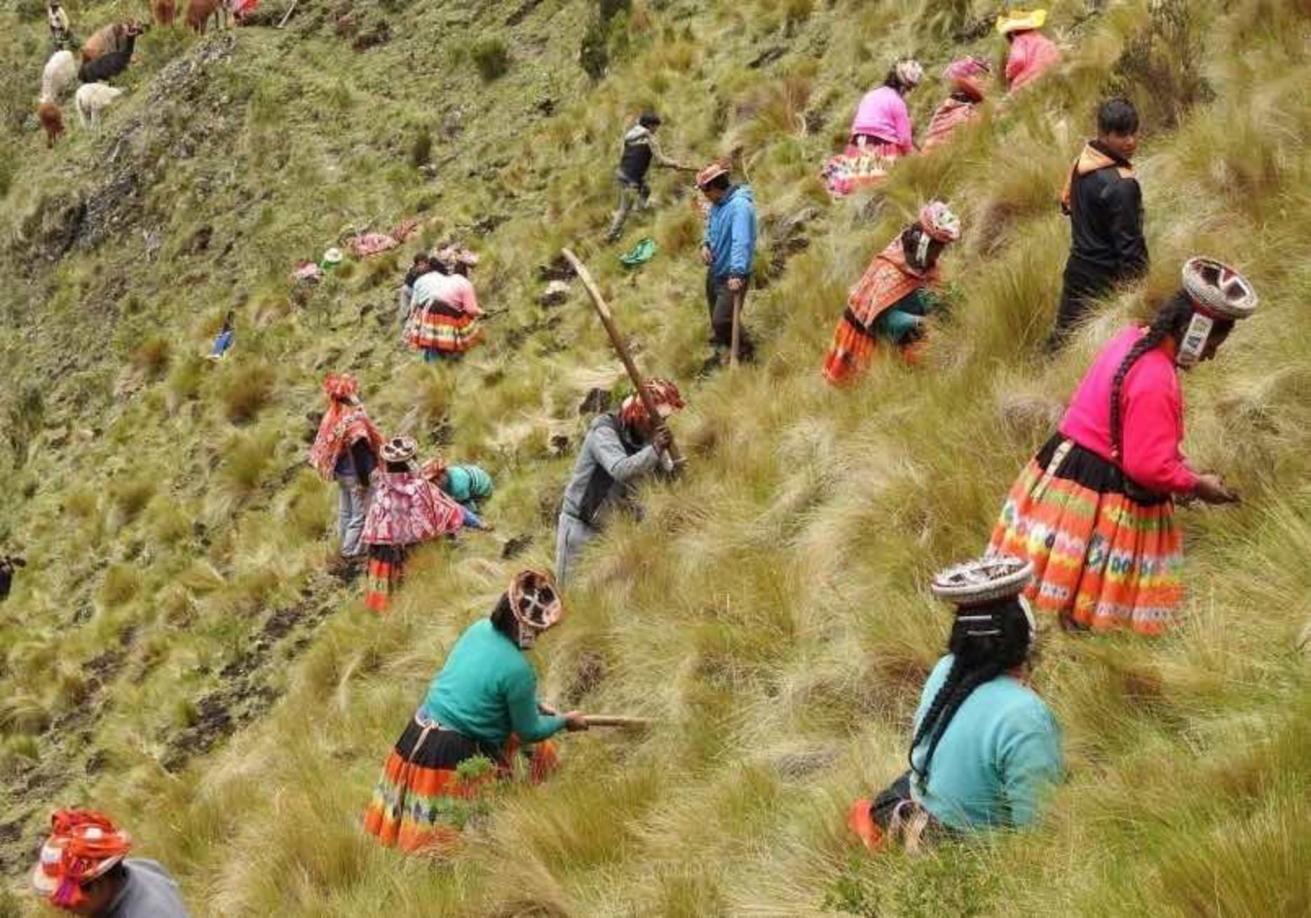
Reforestation with *Polylepis* seedlings by the community of Huilloc, upstream from Marcacocha in the Patacancha Valley. Currently, 680 hectares have been reforested since 2000. Photograph: ECOAN

One such project that has been particularly successful across the region over many years has been implemented by Asociación Ecosistemas Andinos (ECOAN) (Aucca and Ramsay 2005). Founded in 2000, ECOAN works closely with local communities and over the last two decades has planted over 3 million native trees in the Peruvian Andes, including 1.6 million in the high zones of the Cordillera Vilcanota in the Cusco region (Aucca and Ramsay 2005; Cranford and Mourato 2011; Pinos 2020). In the Patacancha Valley alone, the community of Huilloc, located upstream from Marcacocha at 3650 m asl., has worked with ECOAN to reforest 680 hectares of communal land with 231 000 seedlings of queuña (*Polylepis*) (Fig. 6). These habitats, which trap mist and ensure ecosystem services such as providing clean water, are also major centres of biodiversity and endemism for animals and plants alike, including some wild crop relatives (Fjeldså and Kessler 1996). The programme has also incentivised a decreased reliance on growing *Eucalyptus* wood as fuel (and at the same time reduced the pressure on *Polylepis* woodlands) by supplying more efficient wood-burning stoves to these communities (Aucca and Ramsay 2005). This strategy of combining traditional cultural and ecosystem management practices with an acknowledgement of contemporary socio-economic drivers is now enabling ECOAN to help facilitate a broader initiative through Acción Andina (accion-andina.org) to restore a million hectares of *Polylepis* forest and wetland areas across the high Andes.

## Conclusions

The Marcacocha record provides an environmental window on how Andean societies developed and their land-use practices evolved over the last 4000 years in relation to climate change. One of the last chapters in this long history was the vertical migration of both plants and people during a period of warming beginning from *c*. AD 1100, which led to a new zone being developed for efficient agriculture, with resilient watershed management and sustainable agricultural practices being implemented. Practical solutions involving agroforestry, major terracing and other forms of watershed protection, combined with large-scale collection of camelid dung for heating and to sustain soil fertility, and irrigation supplemented by meltwater, were some of the measures that contributed subsequently to the success of maintaining the Inca Empire´s food surpluses.

With the arrival of the Spaniards, a major population decline occurred of both indigenous people and livestock, and the abandonment of many of the sustainable environmental practices and loss of knowledge that had been developed over previous centuries. Nevertheless, new crops, livestock, ploughing methods and management practices were introduced into the Andean rural economy and absorbed, a mark of a resilient culture that adapted pragmatically. However, it is clear that increasingly severe environmentally driven pressures, this time coupled with changing socio-economic pressures, are once again challenging livelihoods across the region.

Highland communities typically have significant social capital (Andolina et al. 2009) and, as evident from the palaeoenvironmental history recorded at Marcacocha, have a long history of adaptation. Indeed, as Young and Lipton (2006; 73) note ‘… *they share an overriding bond of collective action, trust, and community cohesion that supersedes the external authority imposed by regional and national conservation institutions’*. These qualities are at the heart of the capacity of communities to adapt and speak to the fact that, whilst there are multiple strategies available to help communities build resilience, many constraints to implementation remain, including the identification of stakeholders, policy makers and incentives required to both engage and maintain community engagement (e.g. Inbar and Llerena 2000; Llambí et al. 2005; Pramova et al. 2015). Whilst many drivers of (and obstacles to) agricultural sustainability and landscape stability therefore constitute contemporary challenges, it is clear that they nevertheless have a long historical precedent. The most successful recent attempts to build resilience in the region in the face of increasing environmental uncertainty have involved a combination of climate-smart agricultural strategies, coupled with local, community-led initiatives with positive incentives that lean on traditional ecological knowledge. This combined approach then becomes a viable model for improving the livelihoods of many vulnerable highland rural communities in the Andes and indeed, across the globe.

## Supplementary Information

Below is the link to the electronic supplementary material.

Supplementary file1 (PDF 925 KB)
