# Supplementary material for: Trees, terraces and llamas: Resilient watershed management and sustainable agriculture the Inca way
Source: Ambio. 2025 Jan 22;54(5):793–807. doi: 10.1007/s13280-024-02121-5 (PMC11965077; doi:10.1007/s13280-024-02121-5)
Supplement: Supplementary file 1 — Supplementary file1 (PDF 925 KB) [file 13280_2024_2121_MOESM1_ESM.pdf]

Ambio

Supplementary Information

This supplementary information has not been peer reviewed.

Trees, terraces and llamas: resilient watershed management and sustainable agriculture the Inca way

Figure S1. Impacts on the landscape post-conquest: (A) goats feeding on the protective vegetation on the slope between the basin of Marcacocha and the road above; (B) cattle browsing and preventing regeneration in *Polylepis* forest fragment at *c*. 4000 m asl above the community of Huilloc, several kilometres upstream from Marcacocha; (C) planted *Eucalyptus* colonising abandoned Inca terraces in the Patacancha Valley between Marcacocha and Ollantaytambo; (D) unsustainable harvesting of aliso (*Alnus acuminata*), next to the Patacancha river, an important species also for protecting river banks from erosion. All photos: August 1996.

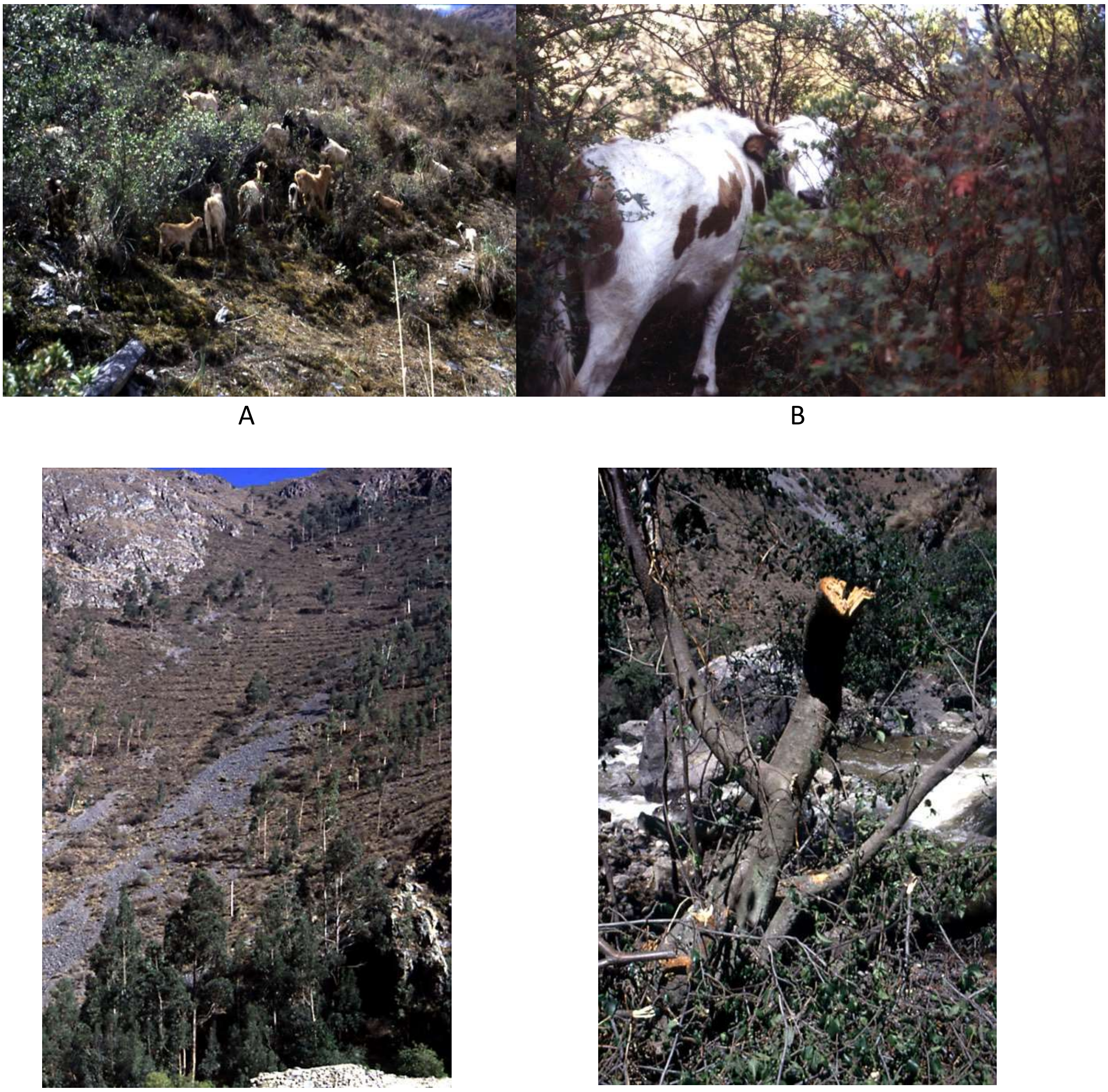

A B

C D

Table S1. Dates from the Marcacocha sequence. Full chronological details are provided in Chepstow-Lusty et al. (2007). The methodology to derive the ^{210}Pb dates followed Flynn et al. (1968). The radiocarbon dates (measured on bulk material) were calibrated using the SHCal04 dataset (McCormac et al. 2004) in conjunction with version 5.0 of the CALIB calibration program (Stuiver and Reimer 1993) and are cited as years before present (CE 1950).

Depth (cm)	Method	Laboratory Reference	^{14}C age (yr BP)	Median calendar date	Calibrated date	
0–2	^{210}Pb			CE 1991		
10–12	^{210}Pb			CE 1958		
18–20	^{210}Pb			CE 1926		
27–29	^{210}Pb			CE 1918		
39–41	^{210}Pb			CE 1907		
47–49	^{210}Pb			CE 1905		
50–51	^{210}Pb			CE 1845		
101–102	^{14}C	Beta-190482	400±40	CE 1540	1σ	CE 1458–CE 1510 (54%)
						CE 1554–CE 1556 (1%)
						CE 1574–CE 1621 (45%)
					2σ	CE 1454–CE 1626 (100%)
110–118	^{14}C	Q-2917	620±50	CE 1360	1σ	CE 1315–CE 1357 (52%)
						CE 1381–CE 1419 (48%)
					2σ	CE 1295–CE 1437 (100%)
210–218	^{14}C	Q-2918	1460±50	CE 630	1σ	CE 568–CE 678 (100%)
					2σ	CE 440–CE 485 (3%)
						CE 537–CE 775 (97%)
310–318	^{14}C	Q-2919	1805±50	CE 280	1σ	CE 138–CE 199 (20%)
						CE 206–CE 403 (80%)
					2σ	CE 30–CE 37 (1%)
						CE 51–CE 543 (99%)
478–486	^{14}C	Q-2920	2245±50	BCE 240	1σ	BCE 384–BCE 160 (95%)
						BCE 133–BCE 117 (5%)
					2σ	BCE 479–BCE 470 (<1%)
						BCE 414–BCE 33 (99%)
						BCE 36–BCE 52 (<1%)
610–618	^{14}C	Q-2921	3650±60	BCE 1960	1σ	BCE 21374–BCE 1754 (100%)
						BCE 2430–BCE 2425 (<1%)
					2σ	BC E2401–BCE 2381 (<1%)
						BCE 2348–BCE 1607 (99%)
						BC~E 1572–BCE 1559(<1%)
						BCE 1548–BCE 1540(<1%)

References

Chepstow-Lusty, A., M.R. Frogley, B.S. Bauer, M. Leng, A. Cundy, K.P. Boessenkool, and A. Gioda. 2007. Evaluating socio-economic change in the Andes using oribatid mite abundances as indicators of domestic animal densities. *Journal of Archaeological Science* 34: 1178–1186.

Flynn, W.W. 1968. Determination of low levels of polonium-210 in environmental materials. *Analytica Chimica Acta* 43: 221–226.

McCormac, F.G., A.G. Hogg, P.G. Blackwell, C.E. Buck, T.F.G. Higham, and P.J. Reimer. 2004. SHCal04 southern hemisphere calibration 0–1000 cal BP. *Radiocarbon* 46: 1087–1092.

Stuiver, M., and P.J. Reimer. 1993. Extended ^{14}C data base and revised CALIB 3.0 ^{14}C age calibration program. *Radiocarbon* 35: 215–230.
